# Effectiveness of video consultations in type 1 diabetes patients treated with insulin pumps in the outpatient clinic: a randomised controlled trial

**DOI:** 10.1007/s00125-025-06585-2

**Published:** 2025-11-07

**Authors:** Anders N. Ø. Schultz, Robin Christensen, Georg Bollig, Kristian Kidholm, Frans Brandt

**Affiliations:** 1 Department of Internal Medicine, University Hospital of Southern Denmark, Aabenraa, Denmark; 2 Centre for Innovative Medical Technology, Odense University Hospital, Odense, Denmark; 3 Department of Regional Health Research, University of Southern Denmark, Sønderborg, Denmark; 4 Research Unit of Rheumatology, Department of Clinical Research, University of Southern Denmark, Odense University Hospital, Odense, Denmark; 5 Section for Biostatistics and Evidence-Based Research, the Parker Institute, Bispebjerg and Frederiksberg Hospital, Copenhagen, Denmark; 6 Department of Anesthesiology, Intensive Care, Palliative Medicine and Pain Therapy, HELIOS Klinikum, Schleswig, Germany; 7 Department of Palliative Medicine, University of Cologne, Faculty of Medicine and University Hospital, Cologne, Germany

**Keywords:** Diabetes mellitus, Insulin pumps, Randomised controlled trial, Telemedicine, Video consultations

## Abstract

**Aims/hypothesis:**

The aim of this work was to assess the effect of video consultations over 1 year compared with usual care for patients with type 1 diabetes treated with insulin pumps, with time in range (TiR) as the primary outcome measure.

**Methods:**

We carried out a 52 week, open label, randomised, controlled superiority trial including adult type 1 diabetes patients treated with insulin pumps. Participants were recruited from the Hospital of Southern Jutland and were adult patients, diagnosed with type 1 diabetes mellitus who had used an insulin pump for at least 6 months. Participants were randomised to video consultations (intervention) or physical consultations (control) using a computer-generated block randomisation sequence in a 1:1 allocation, stratified for sensor type (continuous glucose monitor and flash glucose monitor, respectively).Since this was an ‘open-label’ trial, neither the healthcare professionals providing the treatment nor the participants were blinded to allocation after randomisation. The primary outcome measure was the percentage of TiR (glucose levels 3.9–10.0 mmol/l) from week 51 to 52, measured by continuous glucose monitoring. Continuous endpoints were analysed using ANCOVA, with randomised treatment and stratification groups as fixed effects and the baseline value as a covariate. Missing data in the intention-to-treat (ITT) population were addressed using multiple imputation.

**Results:**

Of the 76 randomised participants (ITT population, 38 per group, median age 49 years, 51% women), 32 participants in the intervention group and 31 in the control group completed the study. Least square means TiR at 1 year was 64.3% in the video group and 63.5% in the control group, with a clinically insignificant difference of 0.8 percentage points (95% CI −5.3, 6.9; *p*=0.25). For secondary outcomes, the video group was superior in terms of treatment satisfaction and reduction in HbA_1c_. However, the video group experienced an inferior impact on quality of life.

**Conclusions/interpretation:**

Video consultations did not significantly improve the primary endpoint. However, compared with control, the intervention was associated with superior treatment satisfaction and a favourable effect on HbA_1c_, albeit with an inferior impact on quality of life.

**Trial registration:**

ClinicalTrials.gov NCT04612933

**Funding:**

The study received funding from Knud and Edith Eriksens Mindefond. The Section for Biostatistics and Evidence-Based Research, the Parker Institute, Bispebjerg and Frederiksberg Hospital is supported by a core grant from the Oak Foundation.

**Graphical Abstract:**

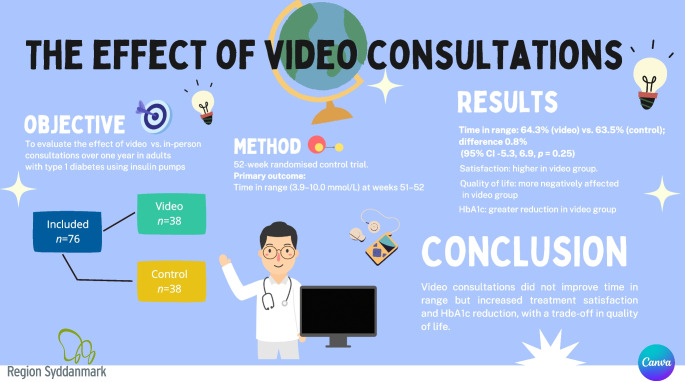

**Supplementary Information:**

The online version of this article contains peer-reviewed but unedited supplementary material available at 10.1007/s00125-025-06585-2.


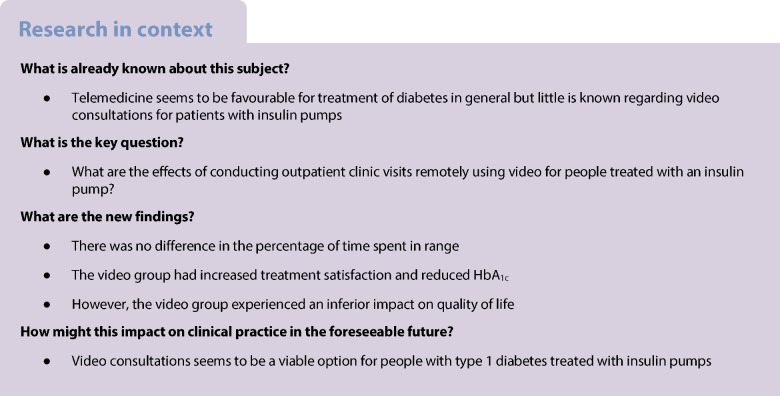


## Introduction

One way of managing type 1 diabetes mellitus is by using insulin pumps for continuous insulin delivery instead of insulin bolus regimens. Research has revealed that centres with a high volume of insulin pump patients experience fewer complications and achieve superior metabolic management as demonstrated by lower HbA_1c_ levels, compared with smaller centres [1]. Nonetheless, patients attending such centres may face challenges, such as increased distance, longer travel times and inconvenience. Consequently, some individuals may forego insulin pump therapy altogether or delay seeking assistance from healthcare providers for pump-related technical difficulties or other medical concerns.

One way to overcome this might be by using telemedicine. Telemedicine has been proven to improve glycaemic management among patients with type 1 diabetes. One systematic review reported that HbA_1c_ was lower in patients who received telemedicine consultations than in those who did not [2]. However, the study included synchronous, asynchronous or a combination of both forms of video consultations [2]. Furthermore, only 9% of the 20 studies assessing HbA_1c_ levels had a low risk of bias. The review only included one study with patients using insulin pumps with synchronous and asynchronous telemedicine modalities [3]. Since patients with insulin pumps have gone through a more intensive education programme, one might speculate that they, in general, have higher technological literacy than patients using multiple daily injections of insulin [4]. Hence, it might be easier for insulin pump patients to share data and conduct video consultations.

When it comes to patients with diabetes treated with insulin pumps, few studies have explored telemedicine’s efficacy [3, 5]. In most of these studies, the telemedicine groups had more scheduled consultations with healthcare professionals (HCPs) than the standard-care groups. In this trial, we intended to compare video consultations with regular in-person consultations, where the number of scheduled interactions between the patient and HCPs remained the same. We focused on glycaemic management as well as addressing more patient-oriented outcomes, such as biopsychosocial impact and treatment satisfaction.

Hence, in this study, our primary objective was to assess the effect of video consultations over 1 year, compared with usual care, on the time in range (TiR; the percentage of time spent at glucose levels of 3.9–10.0 mmol/l) in patients with type 1 diabetes managed with insulin pumps.

## Methods

### Study design, participants and setting

A 52 week, open-label, randomised, controlled superiority trial was conducted, including patients with type 1 diabetes treated with insulin pumps. A detailed protocol for the study has been published [6]. Patients were recruited from the diabetes outpatient clinic of the Department of Internal Medicine at the Hospital of Southern Jutland in Sønderborg. Potential participants were notified of the project by written information posted to them before their planned visit. If interested, they received verbal information about the study in a private room during their visit to the outpatient clinic. The participants were given 48 h to consider whether they would like to participate. Inclusion criteria were as follows: adult patients (>18 years); diagnosed with type 1 diabetes; and using an insulin pump for at least 6 months. Exclusion criteria were as follows: no internet access; not able to adhere to protocol; and not able to speak or read Danish.

Sex was determined from Danish patient records through the CPR (Central Personal Register) number system, in which legal sex is encoded by the parity of the last digit (an odd digit denotes male sex and an even digit denotes female sex).

In Denmark, patients can choose from a range of insulin pumps based on their personal preferences and the recommendations of their healthcare provider. The specific pumps available vary with each tender round, a formal procurement process in which suppliers submit bids based on a predefined set of specifications. Contracts are then awarded to selected suppliers for the duration of the tender period. As a result, a variety of brands and models were included in the study. Despite manufacturer differences, the pumps are comparable in terms of core functionality. Some models in use include features such as low glucose alerts. However, none of the pumps offer fully automated insulin delivery or a closed-loop system.

### Intervention and randomisation

Participants were randomly assigned to receive either video consultations (experimental group) or standard care (control group). Those in the video consultation group continued their usual treatment but aimed to have their appointments (scheduled and unscheduled) conducted via video consultations. The control group received standard care as per routine, attending appointments in person at the outpatient centre. Both groups kept their regular diabetes practitioners, and the appointment frequency was decided by the patient and their practitioner at each visit, with the possibility of patient-initiated visits if needed before the next scheduled appointment. Randomisation was conducted using a pre-specified list of variable block size (two to six participants in each block), an allocation ratio of 1:1, and a stratification for sensor type (continuous glucose monitor and flash glucose monitor, respectively). The randomisation sequences were prepared by a biostatistician with no clinical involvement in the trial (RC). After baseline measurements, the participants were allocated to either video consultations or management as usual (dependent on the list of random numbers). The allocation remained concealed in a password-protected computer file only accessible by the biostatistician and the data manager. However, being an ‘open-label’ trial, neither the HCPs providing the treatment nor the participants were blinded to allocation after randomisation.

### Sample size and power considerations

The power and sample size estimations for this study were based on a Minimal Clinically Important (Target) Difference for TiR of 10% [7]. Hence, a sample size of 100 participants in the intention-to-treat (ITT) population, randomised (1:1 approximately [50 vs 50]), would provide sufficient statistical power (84.2%) to detect a 10% point difference in TiR (i.e. between the groups). Thus, if the effectiveness of video consultations in type 1 diabetes patients treated with insulin pumps corresponds to a 10% TiR improvement compared with management as usual, the trial was designed to be robust against withdrawals corresponding to 10% attrition during the 1 year trial period. This attrition percentage is similar to that in previous studies [3, 8]. However, in case of inability to recruit the target number, a pre-specified date was set at 30 June 2023 for the last patient’s first visit.

### Outcomes and data collection

The primary outcome for this trial was the percentage of time spent in TiR (glucose level 3.9−10.0 mmol/l) at the end of the study, measured in the 2 weeks prior to the end of the study visit (50–52 weeks). Secondary outcomes were divided into biopsychosocial outcomes and treatment satisfaction, and glycaemic management. The biopsychosocial and treatment satisfaction outcomes were as follows: changes in treatment satisfaction measured using the Diabetes Treatment Satisfaction Questionnaire change (DTSQc); and treatment satisfaction measured using the DTSQ status (DTSQs) [9]. Quality of life was measured using Audit of Diabetes Dependent Quality of Life 19 (ADDQoL19) [10]. The questionnaires were distributed to participants at baseline and at the end of study (EOS) visit (DTSQc was only distributed at EOS).

Glycaemic management was measured using the change in HbA_1c_ and percentage of time spent in range: time below range (TbR) level 2 (severe, <3.0 mmol/l); TbR level 1 (moderate, 3.0–3.8 mmol/l); time above range (TaR) level 2 (severe, >13.9 mmol/l); TaR level 1 (moderate, 10.1–13.9 mmol/l); and glycaemic variability. Glycaemic outcomes were collected at baseline and EOS. Glucose sensor data were collected 2 weeks retrospectively after enrolment and 2 weeks before the EOS visit. For each participant, the sensor data had to be active at least 70% of the time for 14 days, as recommended [11].

Study data were collected and managed using REDCap electronic data capture tools hosted at OPEN (Open Patient Data Explorative Network, Department of Clinical Research, University of Southern Denmark; https://www.sdu.dk/da/forskning/open). REDCap (Research Electronic Data Capture) is a secure, web-based software platform designed to support data capture for research studies, providing the following: (1) an intuitive interface for validated data capture; (2) audit trails for tracking data manipulation and export procedures; (3) automated export procedures for seamless data downloads to common statistical packages; and (4) procedures for data integration and interoperability with external sources [12, 13].

### Statistical analysis

Participant demographics and the main analyses are based on the ITT population. Categorical data are reported as counts and proportions, while continuous data are expressed as mean and SD or median and IQR, depending on the data distribution. We did not statistically compare groups at baseline; instead, any noteworthy discrepancies were highlighted in the results based on their clinical relevance.

The ITT principle asserts that the effectiveness of a treatment policy can be best assessed by evaluating the intention to treat (the planned treatment regimen) rather than the actual treatment given [14]. Accordingly, participants allocated to a specific group at baseline were followed up, assessed and analysed as members of that group, irrespective of their adherence to the planned course of treatment (i.e. independent of withdrawals and crossover phenomena) [15]. All 95% CIs and *p* values are two-sided. The 95% CIs were not adjusted for multiplicity and should not be used in place of hypothesis testing. No adjustments for multiplicity were applied [16] but the key secondary outcomes were analysed in a prioritised order (i.e. ‘gatekeeping procedure’) [17]. The analyses of the key secondary endpoints were listed in order under ‘Outcomes and data collection’. The statistical tests were performed and interpreted in sequence until one of the analyses failed to show a statistically significant difference at a statistical significance level of 0.05 [17]. Since a statistically non-significant test is not sufficient to claim ‘no difference’, to show ‘no difference,’ a smallest clinically relevant size of the difference (it might be 0) must be defined. If all clinically relevant differences are excluded from the difference’s CI, a ‘no difference’ or similarity/comparability conclusion is reasonable [15]. Based on our original sample size estimation, we tentatively defined a 95% CI excluding differences greater than 10% in TiR between groups as indicating the absence of a clinically meaningful difference.

Continuous endpoints were analysed using an ANCOVA model, with randomised treatment and stratification groups as fixed effect factors and the baseline endpoint value as a covariate. Missing data were imputed using retrieved participants from the same randomised treatment group, with results combined according to Rubin’s rule [18]. The main analyses are reported as least squares means and SEs, with differences between groups and corresponding 95% CIs adjusted for stratification and baseline outcome measurements. Least squares means, derived from a general linear model with covariate adjustment, provide covariate-adjusted estimates that enhance interpretability and align with the model’s hypothesis tests, enabling more precise and meaningful group comparisons.

In addition to the preplanned analyses stated in the statistical analysis plan, post hoc subgroup analyses for female sex age above 65 years and TiR <70% were also performed, as well as analyses for number of visits and different biopsychosocial outcomes and treatment satisfaction scores.

All data were analysed using R-4.4.2 for Windows and Rstudio 2024.09.1.

## Results

**Fig. 1:**
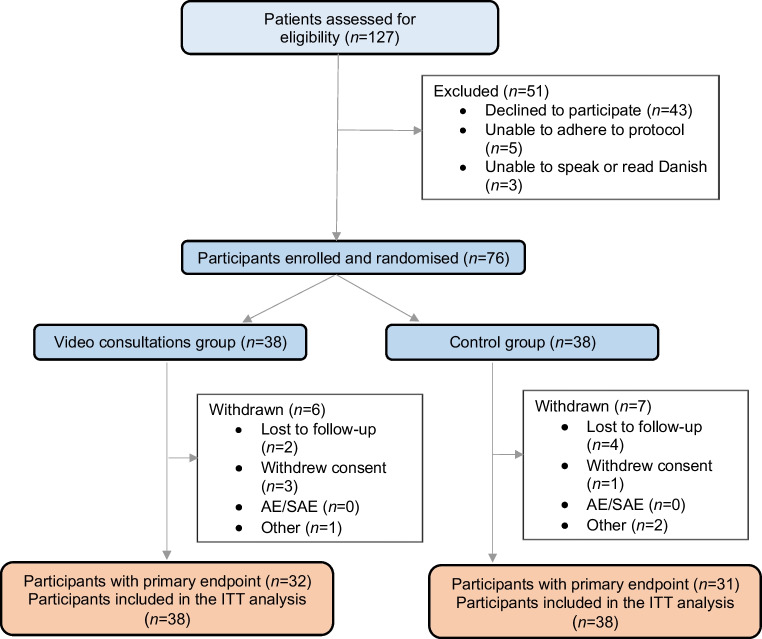
Patient flow throughout the trial. AE, adverse events; SAE, serious adverse events

From June 2021 through June 2023, 127 patients were assessed for eligibility, of whom 119 (94%) were found to be eligible. The trial was stopped due to time limitations, and the pre-specified sample size was not reached. Of the eligible patients, 76 (64%) were included and randomised to video consultations (*n*=38) or standard consultations (*n*=38) (Fig. 1). The median age for included participants was 49 years and 51% were women. There were no clinically relevant discrepancies between the two participant groups at baseline (Table 1).

**Table 1:** Characteristics of participants in the RCT

Characteristic	Video group (*n*=38)	Control group (*n*=38)	Total (*n*=76)
Age, years, median (IQR)	48.5 (17.75)	49.5 (23.75)	49.0 (22.50)
Female sex, *n* (%)	21 (55.3)	18 (47.4)	39 (51.3)
Diabetes duration, years	26.0 ± 14.5	25.9 ± 13.4	25.9 ± 13.9
Weight, kg	85.7 ± 18.7	85.1 ± 17.4	85.4 ± 18.0
BMI, kg/m^2^	28.0 ± 4.92	27.5 ± 6.38	27.8 ± 5.62
Sensor type: continuous glucose monitoring, *n* (%)	31 (81.6)	32 (84.2)	63 (82.9)
Primary outcome			
TiR (3.9–10.0 mmol/l), %	60.7 ± 15.3	61.1 ± 14.2	60.9 ± 14.7
Secondary outcomes			
DTSQs (range 0–36)	31.1 ± 4.24	30.4 ± 4.42	30.7 ± 4.31
ADDQoL19 (range −9 to +3)	−1.14 ± 1.09	−1.84 ± 1.64	−1.51 ± 1.44
HbA_1c_, mmol/mol	60.2 ± 7.16	58.3 ± 7.13	59.3 ± 7.17
HbA_1c_, %	7.66 ± 0.66	7.49 ± 0.65	7.59 ± 0.66
TbR (<3.0 mmol/l), %	0.49 ± 1.60	0.16 ± 0.37	0.33 ± 1.18
TbR (3.0–3.8 mmol/l), %	1.64 ± 2.16	1.23 ± 1.50	1.44 ± 1.87
TaR (>13.9 mmol/l), %	11.40 ± 11.0	10.20 ± 8.59	10.80 ± 9.85
TaR (10.1–13.9 mmol/l), %	26.20 ± 7.68	27.50 ± 8.84	26.08 ± 8.22
Glycaemic variability, %	34.50 ± 7.47	31.90 ± 4.51	33.02 ± 6.23

Values are reported as means ± SD unless otherwise indicated

**Fig. 2:**
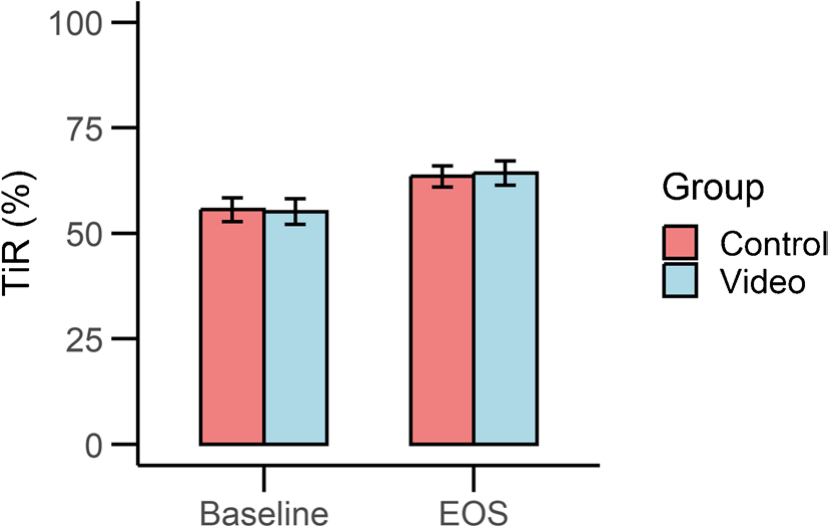
Percentage TiR at baseline and at the EOS from the participants of the control and intervention group (video). Estimates are least squares means and SEs

At the EOS, there was no difference between groups in the primary outcome (Fig. 2). Based on the predefined rule, the findings indicate that the observed difference of 0.8 percentage points (95% CI −5.3, 6.9; *p*=0.25) between the video and control groups does not exceed the clinically meaningful threshold of 10%. Therefore, the results suggest the absence of a clinically meaningful difference in TiR between the groups after 1 year. All secondary outcomes are reported in Table 2. Regarding secondary outcomes, the intervention group showed better treatment satisfaction compared with the controls, with a potential difference of 1.22 points for DTSQc (95% CI −2.51, 4.96) and 1.98 points for DTSQs (95% CI −0.98, 4.93). The intervention group was more affected by their diabetes, with a potential difference in ADDQoL19 of −0.27 (95% CI −1.07, 0.53). Regarding glycaemic control, only HbA_1c_ demonstrated a favourable difference, being lower in the intervention group than in the control group, with a mean difference of −2.01 mmol/mol (95% CI −5.32, 1.29) (−0.18%; −0.49%, 0.12%).

**Table 2:** Primary and secondary endpoints

Endpoint	Video group	Control group	Difference (95%CI)	*p* value
Primary outcome				
TiR (3.9–10.0 mmol/l), %	64.28 ± 2.91	63.49 ± 2.50	0.8 (−5.30, 6.89)	0.251
Secondary outcomes				
DTQSc (range −18 to 18)	10.23 ± 1.61	9.01 ± 1.42	1.22 (−2.51, 4.96)	0.004
DTQSs (range 0–36)	30.06 ± 1.25	28.09 ± 1.13	1.98 (−0.98, 4.93)	<0.001
ADDQoL19 (range −9 to +3)	−1.6 ± 0.33	−1.33 ± 0.29	−0.27 (−1.07, 0.53)	0.004
Change in HbA_1c_, mmol/mol	−4.13 ± 1.43	−2.12 ± 1.38	−2.01 (−5.32, 1.29)	<0.001
Change in HbA_1c_, %	−0.38 ± 0.13	−0.19 ± 0.13	−0.18 (−0.49, 0.12)	
TbR (<3.0 mmol/l), %	0.44 ± 0.18	0.39 ± 0.16	0.05 (−0.34, 0.44)	0.200
TbR (3.0–3.8 mmol/l), %	1.7 ± 0.24	1.46 ± 0.21	−0.2 (−0.71, 0.31)	-
TaR (>13.9 mmol/l), %	8.92 ± 2.29	10.46 ± 1.87	0.35 (−3.04, 3.75)	-
TaR (10.1–13.9 mmol/l), %	24.3 ± 2.31	25.18 ± 1.86	−2.83 (−6.4, 0.74)	-
Glycaemic variability, %	36.86 ± 1.7	36.86 ± 1.44	2.24 (−0.43, 4.90)	-

Estimates are reported as least squares means ± SEs

### Sensitivity analyses and adherence to protocol

No discrepancies were observed between the main findings and those from sensitivity analyses for primary or secondary endpoints (electronic supplementary material [ESM] Tables 1, 2), indicating robustness of the main inferential measures.

The median number of consultations in the video group was three (IQR 4), ranging from 0 to 10. The control group had a median of four (IQR 4) ranging from 1 to 13, with no significant difference between the groups. Although all participants in the video group had at least one in-person consultation, they had fewer (median 1 [IQR 2]; range 1–5) than the control group, with a median of three visits (IQR 2; range 0–9) and a *p* value of 0.002. The median number of video consultations in the video group was one (IQR 2, range: 0–5) vs 0 in the control group (IQR 0; range 0–1) with a *p* value of <0.001 (ESM Table 3).

Post hoc subgroup analyses by TiR, age and sex showed no statistically significant differences between groups for the primary outcome (ESM Table 4).

Correlations between number of contacts in any of the biopsychosocial outcomes and treatment satisfaction scores were weak and not statistically significant in either group (ESM Table 5).

### Serious adverse events

There were three serious adverse events during the study: two in the intervention group; and one in the control group (ESM Table 6). In the intervention group, one participant was admitted to the hospital for diabetic ketoacidosis due to failure of the insulin pump. The two other events (one in each group) were admissions to hospital for hypoglycaemia. All participants made full recoveries after receiving treatment.

## Discussion

In this study, video consultations did not significantly improve the TiR. However, the video group showed not only a better treatment satisfaction and a greater reduction in HbA_1c_ but also a greater negative impact on quality of life. However, although a statistically significant difference was found for several of the secondary outcomes, the difference appeared to be very small and therefore one might speculate whether they might not be clinically relevant. Moreover, while conducting all of the consultations in the video group online was impossible, the participants in this group did have significantly fewer in-person contacts. The total number of contacts was equal between the two groups, suggesting that video consultations can save some in-person consultations while maintaining the same standard of treatment in terms of glycaemic management and even improving treatment satisfaction.

For our study, powered at a 10% difference in TiR, we were able to show no clinically relevant difference between groups. We did find a statistically significant improvement in HbA_1c_, albeit not necessarily clinically relevant, in the video consultation group. According to our knowledge, this is the first study comparing video consultations directly with standard care (in-person consultations) for patients with type 1 diabetes treated with insulin pumps.

Only few studies have looked at TiR as an outcome for telemedicine interventions for patients with insulin pumps. In a recent RCT comparing a hybrid solution using both regular in-person visits and telemedicine vs only face-to-face consultations, the participants using advanced closed-loop systems showed no difference in TiR or any of the other CGM data [19]. These results are similar to those of an observational study looking at the efficacy of telemedicine follow-ups (telephone or video) for sensor-augmented pump therapy (Minimed 640G system) [20]. Similarly to this study, they reported no statistically significant difference in TiR but did find an improvement in HbA_1c_. Another study looking at in-person care before the COVID-19 pandemic vs a hybrid care model (a combination of in-person and virtual care) during the pandemic for people with type 1 diabetes over the age of 65 years found no significant difference in HbA_1c_ but an increase in TiR [21]. However, that study also found a significant increase in visits from 4.2 to 6.3 visits yearly; only around 40% of the participants were insulin pump users. A third study examining the transition from in-person to a virtual multidisciplinary care model (including video and written communication) found an improvement in TiR, HbA_1c_ and TbR [22]. One RCT from 2019, comparing the replacement of two of five in-person visits with telemedicine consultations for patients with insulin pumps to standard in-person care, found no significant difference in HbA_1c_ change [3]. Hence, it seems reasonable to conclude that glycaemic management is as good with video or other telemedicine solutions as it is with in-person consultations for people with an insulin pump.

However, a recent study suggested that it might be possible to reduce the number of contacts for some groups of people with type 1 diabetes and maintain the same level of glycaemic management. The study examined the effects of regular outpatient follow-up visits vs patient-initiated visits [23], where all visits in the 2 year trial period were on-demand and only requested by the patient. In that study, wherein 62 (17%) were insulin pump users, the authors found that patients in the intervention group had fewer consultations during the trial but more telephone consultations than the control group [23]. The study found no significant differences in HbA_1c_.

In terms of patient-reported outcomes, we found increased treatment satisfaction in the intervention group and a greater negative impact on quality of life. Our improved treatment satisfaction is similar to the results from the RCT study that examined telemedicine for people with insulin pumps [3]. In contrast to the RCT, this study indicated that the intervention group reported a more negative impact on quality of life [3]. Similar findings of increased treatment satisfaction were also found in one of the other studies discussed above, while the remaining two did not collect any patient-reported outcomes [22]. In our study, the difference between groups was only estimated to be 0.27 on a scale ranging from −9 to +3. Hence, one might speculate that although statistically different, the finding might not be clinically meaningful; this needs further assessment. Likewise, the RCT examining hybrid delivery for patients with advanced close loop systems found no difference in quality of life [19]. Moreover the post hoc analysis showed no correlation between number of contacts and treatment satisfaction or quality of life, suggesting that both quality of life and treatment satisfaction might be more influenced by other factors than the frequency of contacts.

In terms of adherence, we found that many of the participants in the video consultation group also had in-person consultations. One could speculate that the reasons may be in relation to the patients, healthcare workers or the structural setting. However, our study was not designed to answer this question and therefore we unfortunately do not have data on the reasons why. Semi-structured interviews were conducted with HCPs and patients exploring expectations and experiences with video consultations and will be published separately. However, two qualitative studies exploring people with diabetes and HCPs’ experiences of telemedicine (including telephone and video) consultations in the USA during the COVID-19 pandemic [24, 25] found organisational aspects to be one of the main challenges for proper implementation of telemedicine in the outpatient clinics.

Our study has both strengths and limitations. First, a strength could be that our broad inclusion criteria supported a higher transferability of the results. The mixed use of telemedicine with in-person care in the intervention group makes the findings more generalisable and pragmatic, as this is how most real-world care will be delivered. However, an important limitation of this study is its relatively small sample size, with only 76 participants randomised out of a planned 100. After initiating the trial, we found that the recommendations for minimal clinical important difference for change in TiR in people with diabetes was 5% and not 10% as initially concluded [11]. Hence, since the CI is above 5%, we cannot, with certainty, rule out the possibility of a clinically meaningful difference, for which the study is underpowered. Moreover, the small sample size decreases statistical power, meaning that the CIs around our estimates are wider, especially for secondary and exploratory outcomes, and also increases the risk of type II errors [26]. Therefore, the findings, particularly regarding treatment satisfaction, HbA_1c_ and quality of life, should be interpreted with caution.

Another consideration is that while the study population consisted of adults with type 1 diabetes using insulin pumps from a regional outpatient clinic, reflecting the local demographic in terms of age, regional setting and socioeconomic status ethnicity data were not collected, limiting assessment of representativeness for this factor. While the data might be representative of the clinic population, generalisability to broader populations should be made with caution due to potential regional and ethnic differences.

In conclusion, video consultations did not significantly improve the TiR. However, better treatment satisfaction and a greater reduction in HbA_1c_ were seen, although with a greater negative impact on quality of life. To the best of our knowledge, it seems fair to assume that changing some of the consultations from in-person to video might be possible while still providing sufficient glycaemic management and possibly increasing treatment satisfaction.

## Supplementary Information

Below is the link to the electronic supplementary material.

ESM Tables (PDF 256 KB)

## Data Availability

The datasets generated during and/or analysed during the current study are not publicly available due to the European General Data Protection Regulations but are available from the corresponding author upon reasonable request.

## Abbreviations

ADDQoL19: Audit of Diabetes Dependent Quality of Life 19
DTSQc: Diabetes Treatment Satisfaction Questionnaire change
DTSQs: DTSQ status
EOS: End of study
HCP: Healthcare professional
ITT: Intention-to-treat
TaR: Time above range
TbR: Time below range
TiR: Time in range (glucose levels 3.9–10.0 mmol/l)
