# Supplementary material for: Effectiveness of video consultations in type 1 diabetes patients treated with insulin pumps in the outpatient clinic: a randomised controlled trial
Source: Diabetologia. 2025 Nov 7;69(2):321–9. doi: 10.1007/s00125-025-06585-2 (PMC12779728; doi:10.1007/s00125-025-06585-2)
Supplement: Supplementary file 1 — ESM Tables (PDF 256 KB) [file 125_2025_6585_MOESM1_ESM.pdf]

Electronic supplementary material (ESM)

ESM Table 1: Sensitivity analysis of the primary and secondary outcomes in the per protocol population (informative if Data is Missing Completely At Random)

Primary and secondary endpoints	Video group	Control group	Difference (95%CI)	P-Value
**Clinical and glycaemic outcomes**				
Primary outcome: TiR 3.9–10.0 mmol/L,%)	65.11 ±3.08	61.42±3.18	3.69 (-3.36, 10.75)	0.294
Secondary outcome measures:				
Diabetes Treatment Satisfaction change- range [-18 to 18] Change in Diabetes	9.71 ±2.31	8.41 ±2.31	1.30 (-4.79, 7.39)	0.658
Treatment Satisfaction status–range [0-36]	29.67 ±2.23	29.67 ±2.23	-0.5(-6.46, 5.46)	-
ADDQoL19 –range [-9 to +3]	-1.45 ±0.47	-1.58 ±0.44	-0.12(-1.14, 1.39)	-
Change in HbA1C - mmol/mol	-4.76 ±1.75	-1.33 ±1.89	-3.42(-7.54, 0.69)	-
Change in HbA1C (%), mean (SD)	−0.44 ±0.16	-0.12 ± 0.17	-0.32(-0.65, 0.06)	-
TbR <3.0 mmol/L, %	0.38 ±0.12	0.18 ±0.12	0.21(-0.07, 0.48)	-
TbR 3.0-3.8 mmol/L, %	1.30 ±0.32	1.06 ±0.26	0.24(-0.44, 0.92)	-
TaR >13.9 mmol/L, %	9.70 ±1.80	8.84 ±1.89	0.86(-3.59, 5.30)	-
TaR 10.1-13.9 mmol/L, %	23.06 ±2.81	27.37 ±2.59	-4.31(-11.09, 2.47)	-
Glycaemic variability, %	37.32 ±1.94	35.68 ±2.25	1.64(-2.75, 6.03)	

Estimates are be reported Least Squares Means ±Standard Errors unless otherwise indicated.

ESM Table 2: Sensitivity analysis of primary and secondary outcomes in the Intention to treat population with missing data replaced by baseline observation carried forward (informative is Data is Missing Not At Random)

Primary and secondary endpoints	Video group	Control group	Difference (95%CI)	P-Value
**Clinical and glycaemic outcomes**				
Primary outcome: TiR 3.9–10.0 mmol/L, %	62.15 ±2.25	62.71 ±2.1	-0.56(-5.49, 4.36)	0.819
Secondary outcome measures:				
Diabetes Treatment Satisfaction change- range [-18 to 18] Change in Diabetes	9.95 ±1.82	9.01 ±1.70	0.94(-3.57, 5.45)	0.674
Treatment Satisfaction status–range [0-36]	30.3 ±0.77	30.24 ±0.78	0.06(-1.78, 1.89)	-
ADDQoL19 –range [-9 to +3]	-1.5 ±0.14	-1.55 ±0.15	0.05(-0.3, 0.4)	-
Change in HbA1C - mmol/mol	-2.49 ±1.13	-1.18 ±1.14	-1.31(-4.02, 1.4)	-
Change in HbA1C (%)	-0.23±0.10	-0.11 ±0.10	-0.12(-0.37, 0.13)	-
TbR <3.0 mmol/L, %	0.35 ±0.14	0.31 ±0.13	0.03(-0.27, 0.34)	-
TbR 3.0-3.8 mmol/L, %	1.26 ±0.19	1.18 ±0.17	0.07(-0.34, 0.48)	-
TaR >13.9 mmol/L, %	9.65 ±1.23	8.62 ±1.12	1.04(-1.54, 3.62)	-
TaR 10.1-13.9 mmol/L, %	24.69 ±1.54	27.07 ±1.39	-2.38(-5.75, 1.00)	-
Glycaemic variability, %	34.59 ±0.98	32.91 ±1.01)	1.67(-0.46 - 3.81)	

Estimates are be reported Least Squares Means ±Standard Errors unless otherwise indicated.

ESM Table 3: Number of contacts during the study.

	Video group	Control group	Total	P value
Video	1 (2)[0 - 5]	0 (0) [0, 1]	0 (1)[0 - 5]	< 0.001
In-person contacts	1 (2) [1 - 5]	3 (2) [0 - 9]	2 (2.57) [0 - 9]	0.002
Telephone	0 (1) [0 - 4]	1(2) [0, 6]	1(2)[0 - 6]	0.284
Total	3(4) [0 - 10]	4 (4)[1, 13]	4(4) [1- 13]	0.333

Numbers are reported in median, (interquartile range) and [range]

P values are calculated using poisson regression, adjusting for time in the study.

ESM Table 4: Subgroup analyses of Tim-in-Range, Age and gender.

**Subgroup: Time-in-range ≥ 70%[a]**	**Video group**	**Control group**	**Difference (95%CI)**
**Primary outcome:** percentage of time spent in TiR 3.9–10.0 mmol/L (%)	72.86 ±4.41	74.84 ±4.49	-1.97 (-15.94, 11.99)
Secondary outcome measures:			
Diabetes Treatment Satisfaction change- range [-18 to 18]	9.15 ±2.82	6.87 ±4.36	2.28 (-9.65, 14.22)
Change in Diabetes Treatment Satisfaction status–range [0-36]	27.43 ±1.76	25.37 ±2.72	2.06 (-5.57, 9.68)
ADDQoL19 –range [-9 to +3]	-1.6 ±0.52	-2.26 ±0.82	0.66 (-1.68, 3.00)
Change in HbA1C - mmol/mol	-0.18 ±1.39	-1.05 ±1.40	0.86 (-3.5, 5.23)
Change in HbA1C (%)	-0.02±0.13	-0.10 ±0.13	0.08 (-0.32, 0.48)
**Subgroup: Time-in-range < 70%**	**Video group**	**Control group**	**Difference (95%CI)**
**Primary outcome:** percentage of time spent in TiR 3.9–10.0 mmol/L (%)	61.26 ±3.14	59.6 ±2.58	1.66 (-5.44, 8.76)
Secondary outcome measures:			
Diabetes Treatment Satisfaction change- range [-18 to 18]	10.47 ±1.88	8.79 ±1.43	1.68 (-2.67, 6.03)
Change in Diabetes Treatment Satisfaction status–range [0-36]	30.67 ±1.47	28.24 ±1.15	2.43 (-1.01, 5.87)
ADDQoL19 –range [-9 to +3]	-1.74 ±0.40	-1.28 ±0.3	-0.46 (-1.41, 0.49)
Change in HbA1C - mmol/mol	-5.92 ±1.96	-2.04 ±1.63	-3.88 (-8.37, 0.62)
Change in HbA1C (%)	-0.54 ±0.04	-0.19 ±0.15	-0.36(-0.77,0.06)
**Subgroup: Age ≥ 65 years[a]**	**Video group**	**Control group**	**Difference (95%CI)**
**Primary outcome:** percentage of time spent in TiR 3.9–10.0 mmol/L (%)	69.61 ±8.06	68.39 ±8.06	1.22 (-28.09, 30.53)
Secondary outcome measures:			
Diabetes Treatment Satisfaction change- range [-18 to 18]	16.13 ±2.97	9.8 ±2.97	6.33 (-5.32, 17.99)
Change in Diabetes Treatment Satisfaction status–range [0-36]	32.6 ±3.52	29.13 ±3.52	3.47 (-12.84, 19.77)
ADDQoL19 –range [-9 to +3]	-1.42 ±0.54	-1.6 ±0.54	0.17 (-2.31, 2.66)
Change in HbA1C - mmol/mol	-4.94 ±4.66	-2.06 ±4.66	-2.89 (-20.39, 14.61)
Change in HbA1C (%)	-0.45 ±0.43	-0.19 ±0.43	-0.26 (1.86, 1.34)
**Subgroup: Age < 65 years[a]**	**Video group**	**Control group**	**Difference (95%CI)**
**Primary outcome:** percentage of time spent in TiR 3.9–10.0 mmol/L (%)	63.45 ±2.93	62.76 ±2.49	0.69 (-5.73, 7.11)
Secondary outcome measures:			
Diabetes Treatment Satisfaction change- range [-18 to 18]	9.47 ±1.66	8.98 ±1.45	0.49 (-3.48, 4.46)
Change in Diabetes Treatment Satisfaction status–range [0-36]	29.77 ±1.28	28.12 ±1.16	1.65 (-1.49, 4.80)
ADDQoL19 –range [-9 to +3]	-1.6 ±0.36	-1.36 ±0.31	-0.24 (-1.15, 0.67)
Change in HbA1C - mmol/mol	-3.98 ±1.44	-2.11 ±1.40	-1.87 (-5.39, 1.65)
Change in HbA1C (%)	-0.36 ±0.13	-0.19 ±0.13	-0.17 (-0.49, 0.15)
**Subgroup: Gender, female**	**Video group**	**Control group**	**Difference (95%CI)**
**Primary outcome:** percentage of time spent in TiR 3.9–10.0 mmol/L (%)	64.11 ±4.48	64.78 ±3.64	-0.67 (-9.69, 8.36)
Secondary outcome measures:			
Diabetes Treatment Satisfaction change- range [-18 to 18]	10.43 ±2.69	9.77 ±2.08	0.66 (-5.32, 6.64)
Change in Diabetes Treatment Satisfaction status–range [0-36]	32.52 ±1.41	28.4 ±1.10	4.12 (0.85, 7.39)
ADDQoL19 –range [-9 to +3]	-1.83 ±0.49	-1.59 ±0.32	-0.24 (-1.34, 0.85)

Change in HbA1C - mmol/mol<br>Change in HbA1C (%)	-1.23 ±1.91<br>-0.11 ±0.17	-0.94 ±1.71<br>-0.08 ±0.16	-0.29 (-4.60, 4.02)<br>-0.03 (-0.42, 0.37)
**Subgroup: Gender, Other**	**Video group**	**Control group**	**Difference (95%CI)**
**Primary outcome:** percentage of time spent in TiR 3.9–10.0 mmol/L (%)	65.61 ±3.91	61.34 ±3.54	4.27 (-4.91, 13.46)
Secondary outcome measures:			
Diabetes Treatment Satisfaction change- range [-18 to 18]	10.2 ±2.01	7.98 ±2.01	2.22 (-3.04, 7.48)
Change in Diabetes Treatment Satisfaction status–range [0-36]	28.6 ±1.95	27.72 ±2.06	0.88 (-4.45, 6.20)
ADDQoL19 –range [-9 to +3]	-1.44 ±0.49	-1.1 ±0.51	-0.33 (-1.67, 1.00)
Change in HbA1C - mmol/mo	-7.06 ±2.11	-3.05 ±2.16	-4.01 (-9.37, 1.34)
Change in HbA1C (%)	-0.65 ±0.19	-0.28 ±0.20	-0.37 (-0.86, 0.12)
Estimates are be reported Least Squares Means ±Standard Errors unless otherwise indicated.<br>[a]CGM removed as covariate (only 1 level)			

ESM Table 5: Correlation between number of contacts and biopsychosocial outcomes and treatment satisfaction scores

Score	Video group Correlation [95% CI]	Control group Correlation [95% CI]
Diabetes Treatment Satisfaction change- range [-18 to 18] Change in Diabetes Treatment Satisfaction status–range [0-36]	-0.227 [-0.62, 0.25]<br>-0.018 [-0.48, 0.46]	0.152 [-0.29, 0.54]<br>0.119 [-0.34, 0.54]
ADDQoL19 –range [-9 to +3]	-0.201 [-0.63, 0.32]	0.066 [-0.39, 0.49]

ESM Table 6: Adverse events

Harms and Adverse events	Video group	Control group	Risk Difference (95%CI)	Risk Ratio (95%CI)
Withdrawals due to Adverse Events, no. (%)	0	0	0	0
Prespecified events of interest:				
Episode ofsevere hypoglycemia[a], no. (%)	1	1	-0.0 (-0.072 - 0.072)	1 (0 - 21011018)
Hospital admission for diabetic ketoacidosis, no. (%)	1	0	0.026 (-0.025 - 0.077)	Inf (NaN , Inf)
Serious Adverse Events, no. (%)	2	1	0.026 (-0.061 - 0.114)	2 (0 - 1186495)
MACE, no. (%)	0	0	0	0
- Cardiovascular death, no. (%)	0	0	0	0
- Nonfatal myocardial infarction, no. (%)	0	0	0	0
- Nonfatal stroke, no. (%)	0	0	0	0
Mortality, no. (%)	0	0	0	0

[a]needing assistance from others, but not necessarily hospitalization.
